# Effect of Magnetic Field on Maskless Localized Electrodepositing Three-Dimensional Microstructure of Nano Nickel Crystals

**DOI:** 10.3390/ma17020386

**Published:** 2024-01-12

**Authors:** Menghua Wu, Bingchun Jiang, Yuqing Xiao, Weiping Jia

**Affiliations:** 1 School of Mechanical and Electrical Engineering, Guangdong University of Science and Technology, Dongguan 523083, China; wmh005@163.com (M.W.); jiangbingchun_2008@163.com (B.J.); 2 School of Mechanical Engineering, Dalian University, Dalian 116622, China; uing159369@163.com

**Keywords:** magnetic field, maskless localized electrodeposition, nanocrystalline nickel, three-dimensional microstructures

## Abstract

In the intricate process of maskless localized electrodeposition (MLED) for fabricating three-dimensional microstructures, specifically nickel micro-columns with an aspect ratio of 7:1, magnetic fields of defined strength were employed, oriented both parallel and anti-parallel to the electric field. The aim was to achieve nanocrystalline microstructures and elevated deposition rates. A detailed comparative analysis was conducted to examine the volumetric deposition rate, surface morphology, and grain size of the MLED nickel crystal 3D microstructures, both in the absence and presence of the two magnetic field directions, facilitated by a self-assembled experimental setup. The results indicate that the anti-parallel magnetic field significantly boosts the volumetric deposition rate to a notable 19,050.65 μm^3^/s and refines the grain size, achieving an average size of 24.82 nm. Conversely, the parallel magnetic field is found to enhance the surface morphology of the MLED nickel crystal 3D microstructure.

## 1. Introduction

With the surging demand for metal microstructures across various domains, the innovation in design, processing of 3D metal microstructures, and the development of preparation technologies boasting high process flexibility is experiencing active advancement. As a result, unconventional machining processes, particularly electrochemical micro-machining processes, are garnering attention in MEMS [1], integrated circuit sensors [2,3], microelectronics [4,5], and additional fields [6]. This research is also very important for new very precise and highly sensitive quartz sensors for magnetic measurement which have been developed, and which take temperature compensation into account [2,3]. Over the years, a plethora of electrochemical fabrication methodologies has emerged. Jet plating initially marked a significant stride towards selective electrodeposition processing, though its localization remains suboptimal due to reliance solely on liquid flow for limiting the deposition area [7,8]. Localized electrochemical deposition (LED), for instance, finds frequent application in the deposition of mainly 3D microstructures [9], albeit with prevalent issues of high porosity and subpar surface finish [10,11]. Meanwhile, the meniscus-confined electrodeposition (MCED) process typically takes shape via a micropipette featuring a micron/submicron-size dispensing tip [12]. However, this MCED process is acknowledged for its operational complexity, necessitating new calibrations prior to every experimental endeavor [13].

Thus, the introduction of maskless localized electrodeposition (MLED) is noted in this paper. Characterized as a rapidly advancing microfabrication process, MLED demonstrates proficiency in crafting three-dimensional microstructures from metals such as copper, nickel, and gold, all within an ambient environment, and all without the necessity for intricate tooling [14]. Beyond the technical and economic merits inherent to plating technology, MLED stands as a contributor to reduced development durations and enhanced economic returns [15], particularly in the realms of post-processing and rapid prototyping applications [16,17]. Presently, the majority of investigative efforts surrounding MLED concentrate on understanding how varying parameters influence deposition rates and the characteristics of deposition structures under singular action fields, leaving the results and microstructural characteristics stemming from multi-action field depositions less explored [18]. In view of this, the goal of the paper is to investigate the effect of magnetic field on maskless localized electrodepositing three-dimensional Microstructure of nano nickel crystals.

However, over time, the limitations of electrochemical 3D fabrication methods restricted to a single field of action became apparent. Since the 1970s, experimentation with the incorporation of magnetic fields into the electrodeposition process has been undertaken, leading to a unique set of phenomena in the deposition process and capturing the attention of numerous researchers worldwide. Progress in this area transformed the intervention of magnetic fields into a comprehensive new technology, spanning electromagnetics, electrochemistry, and materials science, and giving rise to a new interdisciplinary domain: magneto-electrochemistry [19,20]. Compared with conventional processes, the application of a magnetic field to electrodeposition offers advantages, enhancing the deposition rate and influencing the surface and microstructure of the deposited material [21,22]. Hence, in delving into the operational variables of the MLED process, particular emphasis has been placed on understanding the impact of magnetic field application, aiming to elucidate its effects on the deposition rate, surface morphology, and grain size of three-dimensional microstructures in this paper.

In this paper, the implementation of a magnetically controlled MLED technique is put forward. This innovative method will utilize non-contact energy transfer to effectively promote localized electrodeposition for the fabrication of metal 3D microstructures. The approach negates the necessity for mask preparation, relying solely on cyclic electrolyte connections; herein, miniature anodes and cathodes act as electrochemical reaction cells, while dynamic anodes and magnetic fields serve as instrumental tools in directing the course of metal electrodeposition. Comprehensive understanding of the working mechanism and the formation process is achieved through a combination of simulation and experimental efforts, entailing meticulous observation and analysis of both macroscopic and microscopic structural features.

## 2. Working Principle of MLED under Action of Magnetic Field

**Figure 1:**
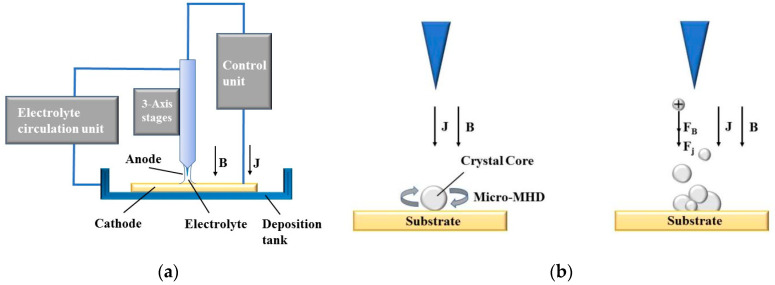
Principle of MLED under action of magnetic field; (**a**) diagram of working principle of MLED; (**b**) example of magnetron nucleation influence.

The working principle of MLED under action of magnetic field is shown in Figure 1. In Figure 1a, during the deposition process, an electric potential is established between the electrode tip and the substrate, mediated by the electrolyte. This applied potential induces a Faraday current, denoted as i_tip_, navigating through the electrolyte and creating a highly localized electric field between the micro electrode tip, poised at a positive potential (anode), and the conducting substrate, positioned at a negative potential (cathode). Consequently, metal ions (*M^+^*) situated near the localized electric field and in contact with the substrate surface undergo reduction. The plating solution, transported via a peristaltic pump, ensures a steady supply of metal ions (*M^+^*), facilitating their rapid deposition onto the substrate surface, directly beneath the tip. This process results in the formation of a dynamic and stable electrolytic column, the dimensions of which can be tailored to satisfy specific application requirements.

Divergent from conventional electroplating techniques, the deposition mechanism depicted in Figure 1a exhibits a high degree of localization, confined primarily to the region directly beneath the electrode tip, with a scope approximately equivalent to the tip’s dimensions [12]. In theory, provided that the anode maintains electrical continuity with the substrate, maneuvering the microelectrode tip relative to the formative surface can yield structures of any conceivable geometry.

In traditional electrochemical deposition, macroscopic magnetohydrodynamic (Macro-MHD) effects typically result in a dominating Lorentz force [19]. This prevalence stems from the consideration of magnetic field introduction as a ‘stirring’ mechanism in conventional approaches, aiming to alleviate differential polarization and mitigate nanoparticle agglomeration [23]. Contrastingly, in the realm of MLED technology, the cyclical injection of plating solution addresses these issues, repurposing the magnetic field’s role to ‘shape control’. Guiding the placement of metal ions and atoms during deposition through magnetic intervention allows for precise manipulation of the internal structure and geometry of the deposit.

Figure 1b presents an example of the influence of magnetron nucleation during MLED under the influence of a magnetic field. It provides a schematic representation of the nucleus being influenced by the micro-MHD effect during the MLED process, and illustrates the processes of ion stressing and polymerization.

In electrochemical systems, the para-magnetism or anti-magnetism of a substance influences the concentration gradient proximate to the electrode, inducing convection in the vicinity. This process is mainly influenced by the magnetization force *F_M_*, and the relevant Equation (1) of *F_M_* is shown below.

(1) $$F_{M}=\frac{1}{2\mu_{0}}\chi_{sol}\nabla B^{2\;}$$

where $F_{M}$ is the magnetization force, $\mu_{0}$ is the vacuum magnetic permeability (4 × Pi × 10^−7^ Vs/Am), ∇B denotes the gradient of magnetic induction, and $\chi_{sol}$ is the magnetization of the solution. Throughout the experiments documented in this paper, *Ni*, a paramagnetic substance, serves as the subject for further investigation. The Equation (2) describing magnetization force’s impact on *Ni* is shown below [20].

(2) $$\nabla \times F_{M}=\frac{\chi_{mol,para}}{2\mu_{0}}\left(\nabla c_{para}\right)\times \left(\nabla B^{2}\right)$$

where ∇ is the differential algorithm, $\chi_{mol,para}$ and $c_{para}$ are the molar magnetization rate and concentration of *Ni*. Consequently, the induction of a magnetic field imparts both microscopic and macroscopic magnetic properties to the electrodeposited structures.

## 3. Experimental

### 3.1. Experimental Set of MLED

**Figure 2:**
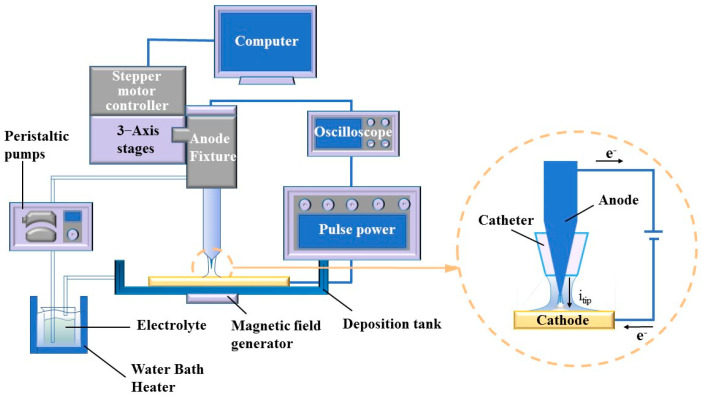
Diagram of MLED experimental set.

The experimental setup, devised specifically for MLED, encompasses an oscilloscope (GDS-1104E, Xinjinshi Technology Co., Ltd., Shenzhen, China), deposition unit, magnetic field generator, electrolyte temperature circulation unit, and a control unit, as illustrated in Figure 2. The deposition unit comprises a copper cathode, a platinum anode, and a catheter, which aligns concentrically with the cone-shaped platinum anode, channeling and directing the electrolyte flow. The platinum anode connects to the positive terminal of the power supply, while the copper cathode links to the negative terminal. The anode-related segment of the deposition unit interfaces with the control unit, facilitating numerical control over the anode’s positioning and movement through computer software. Components of the control unit include 3—Axis stages (ETS-100RG, Sanying Precision Control Instrument and Equipment Co., Ltd., Tianjin, China), stepper motor controller (PMC400-3, Sanying Precision Control Instrument and Equipment Co., Ltd., Tianjin, China), a computer, and pulse power (GKDMZ, Star Tongli Power Supply Equipment Co., Ltd., Chengdu, China), with the ability to adjust the high peak current density on the pulse power, ensuring timely replenishment of deposited ions at the cathode-solution interface. Meanwhile, the electrolyte temperature circulation unit comprises a peristaltic pump (BT100M, Creative Pump Co., Ltd., Baoding, China) and a water bath heater (H2L, Jiangsu Xinchunlan Scientific Instrument Co., Ltd., Changzhou, China).

**Figure 3:**
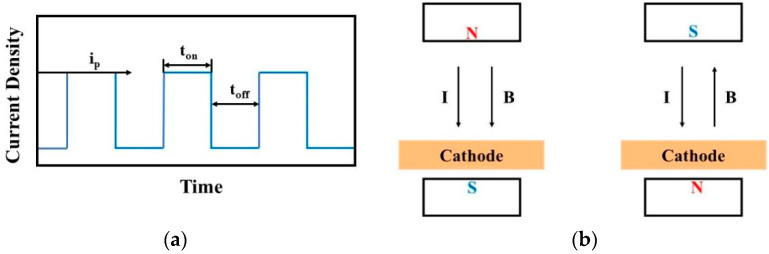
Schematic diagram of pulsed waveform and magnetic pole setting; (**a**) pulse waveform of electrodeposition; (**b**) manner of magnetic pole setting.

The oscilloscope facilitates the visualization of waveforms under various pulse parameters, with the pulse on time (t_on_) and pulse off time (t_off_) adjustable during experimentation. This adjustability enables control over grain size and deposition rate to a certain extent through the observation of waveforms displayed on the oscilloscope. In parallel, to delve into the impact of magnetic control on the deposition process in MLED technology, Nd2Fe14B permanent magnets (K&J Magnetics, Inc., Pipersville, PA, USA) with a magnetic field strength of approximately 0.3 T were strategically placed above the anode and below the copper cathode. This arrangement results in the formation of magnetic lines of force that are nominally “parallel” to the direction of the current flow. Adjustments to the relationship between the N or S poles and the working electrode yield configurations for both parallel and anti-parallel magnetic fields relative to the direction of the electric field, thus enabling subsequent macroscopic and microscopic investigations. Schematic representations of the pulse waveform and the magnetic pole setup for MLED are presented in Figure 3.

### 3.2. Experimental Materials and Methods

The substrate utilized comprised a polished copper sheet with dimensions of 10 mm × 30 mm × 1 mm. For the electrode surface, a sequential polishing process was implemented, utilizing silicon carbide papers of successive grits: 800, 1000, 1500, and 2000. To achieve a smooth and mirror-like surface, a final fine polishing was conducted using 1.0 μm diamond paste on a nylon cloth. Subsequently, the polished copper sheet underwent a pre-treatment process, encompassing alkaline washing (OP-200), etching, and a 0.5 M sulfuric acid wash, all aimed at eliminating oil stains and attachments from the surface of the copper sheet.

The micro-nickel column underwent electrodeposition preparation in a 500 mL bath, comprising the main salt nickel chloride (NiCl_2_·6H_2_O), along with crystal modifiers ammonium chloride (NH_4_Cl) and boric acid (H_3_BO_3_). The electrolyte formulation included 1.2 M of nickel amino-sulfonate (Ni(NH_2_SO_3_)_2_·4H_2_O), 3 M of NiCl_2_·6H_2_O, and 3 M of H_3_BO_3_, all without any additives. Above chemical reagents were purchased from Yi en Chemical Technology Co., Ltd. (Shanghai, China). In the experimental process, Ni(NH_2_SO_3_)_2_·4H_2_O served as the primary supplier of *Ni* ions, NiCl_2_·6H_2_O enhanced electrical conductivity, and H_3_BO_3_ is utilized to regulate the pH value. The electrolyte temperature remained controlled at 323 ± 2 K.

The anode, shaped as a cone, boasts a minimum end diameter of 15 μm. The electrolyte, maintained at a constant temperature, flows from the gap between the anode and the catheter to the cathode, ensuring comprehensive conduction and deposit growth. Controlled via computer, the platinum needle (anode) traverses in relation to the cathode, adhering to a predetermined path trajectory and speed set by the program. During the MLED process, a constant polar spacing is upheld. As the deposit grows and bridges the gap between the tip and the substrate, direct contact occurs between the tip and the growing deposit end, resulting in a sudden surge in current. This prompts the electrode to retract from the deposit end, following a trajectory that aligns with the desired geometry.

## 4. Results and Discussion

### 4.1. Effect of Magnetic Field on Deposition Rate of MLED

**Figure 4:**
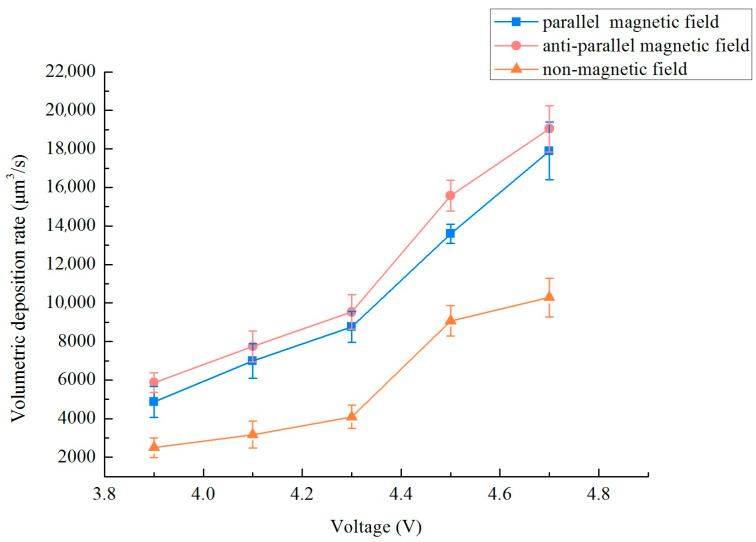
Effect of different magnetic field directions on the volumetric deposition rate.

In the previous research on localized electrodeposition of nanocrystalline coatings, relevant experimental parameters such as electrolyte composition, different magnetic field strengths (0–0.3 T), duty cycles and frequency were applied. The results showed that a magnetic field strength of 0.3 T, a frequency of 10 kHz, and a duty cycle of 0.3 matched well with the localized electrodepositing process. As the research content of this paper is based on the previous research foundation and experimental platform, so to examine the impact of varied magnetic field orientations on the deposition rate of a micro-nickel column crafted utilizing the MLED technique, settings were established at a magnetic field strength B = 0.3 T, a frequency of 10 kHz, and a duty cycle of 0.3. For this experiment, modifications were applied solely to the voltage magnitude and the magnetic field direction. The method of calculating the deposition rate: Using a digital microscope (OLYMPUS, DSX1000, Puhe Photoelectric Technology Co., Ltd., Shanghai, China) to measure the multi-point diameter and height of a micro-nickel column, writing a MATLAB (R2021b) program, treating the micro-nickel column as countless cylinders by using differential method, and then calculating the volume deposition rate by inputting the measured multi-point diameter and height. Figure 4 delineates the influence of disparate magnetic field directions on the volumetric deposition rate of the micro-nickel column. The incorporation of a magnetic field in both directions serves to substantially augment the volumetric deposition rate of the micro-nickel column. Furthermore, it is observed that as the voltage increases, so too does the enhancement of the volumetric deposition rate. Notably, the anti-parallel magnetic field (i.e., aligned in opposition to the electric field direction) demonstrates a more pronounced effect on the rate enhancement, achieving the highest volume deposition rate at 19,050.65 μm^3^/s.

The following reaction equations are mainly involved in the main process of MLED deposition.

(3) $${Ni}^{2+}+2e^{-}\to Ni$$

(4) $$H^{+}+e^{-}\to H_{ad}$$

(5) $$H_{ad}+H_{ad}\to H_{2}↑$$

(6) $$G_{M}=\frac{x_{V}B^{2}}{2\mu_{0}}$$

where ${Ni}^{2+}$ is the nickel ion, $e^{-}$ is the electron, Ni is the nickel atom, $H^{+}$ is the hydrogen ion, $H_{ad}$ is the adsorbed hydrogen atoms, $H_{2}$ is the hydrogen molecule, $G_{M}$ is the magnetic Gibbs free energy, $x_{V}$ is the volumetric magnetization of the material, B is the magnetic induction intensity, and $\mu_{0}$ is the vacuum magnetization. The addition of external magnetic field itself affects the physicochemical reaction and microstate of deposition (including heat of reaction, reaction rate, activation energy, entropy, etc.) [15]. Reaction (3) induces a depletion of *Ni*^2+^ in the microregion; however, the implementation of a magnetic field may facilitate the migration of *Ni*^2+^ ions towards the cathode surface, mitigating the cathodic polarization phenomenon. Gibbs free energy of deposited *Ni* and reduced *H* is one major factor to influence the chemical potential [24]. It is necessary that enhancing the Gibbs free energy difference to reduce the degree of cathodic polarization and promote the reaction of electrodepositing reduction on the cathode surface [25]. Concurrently, the application of an external magnetic field during MLED enhances the magnetic Gibbs free energy difference for reactions (3)–(6), resulting in a more negative value. This shift leads to a reduction in the total energy of the system’s products, thereby fostering the progression of the reaction [9,26].

**Figure 5:**
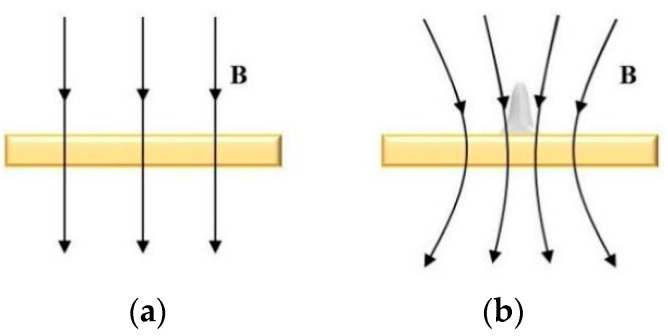
Distribution of basal magnetic field lines (**a**) before micro-nickel column growth; (**b**) after micro-nickel column growth.

The cathode substrate upon which deposition occurs comprises paramagnetic material *Cu*, while the deposited micro-nickel pillar is a weakly magnetic material susceptible to easy magnetization. Consequently, the distribution of magnetic field lines undergoes alteration before and after the growth of the micro-nickel pillar under the influence of a parallel magnetic field, as depicted in Figure 5. As the deposited nickel pillars become magnetized within the magnetic field, the magnetic properties intrinsic to the nickel pillars cause a relative concentration of magnetic field lines at the substrate’s edge, forming a high-density magnetic field region centered around the micro-nickel columns. This phenomenon results in a micro-MHD effect that alters the trajectory of *Ni*^2+^ ions on a microscopic scale, hastening both mass and charge transfer processes within the electrodeposition procedure [19]. Concurrently, *Ni*, being a paramagnetic particle, migrates along the magnetic field lines, influencing the arrangement of grains and the structure of crystals through a magnetic orientation effect. Fundamentally, this magnetic orientation effect dictates that the magnetized grains align according to the direction of minimal magnetization energy at a microscopic level. The direction of the inverse electric field magnetic field aligns with the growth direction of the micro-nickel column, prompting metal ions to continually move and deposit along the growth direction under magnetic force. This dynamic results in a higher volume deposition rate for the inverse electric field direction magnetic field compared with the downward electric field direction magnetic field (i.e., parallel magnetic field).

### 4.2. Effect of Magnetic Field on Microscopic Surface Morphology of MLED 3D Microstructure

**Figure 6:**
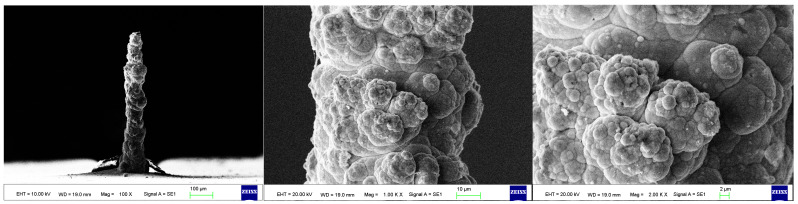
Surface morphology of micro-nickel column fabricated without magnetic field.

**Figure 7:**
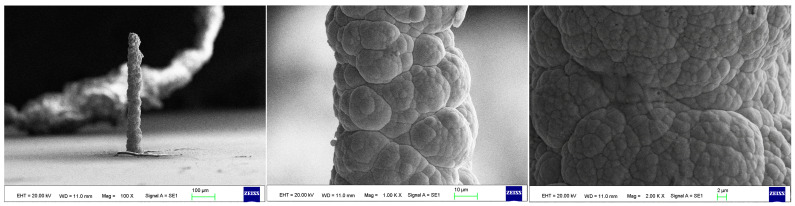
Surface morphology of micro-nickel column fabricated under parallel magnetic field.

**Figure 8:**
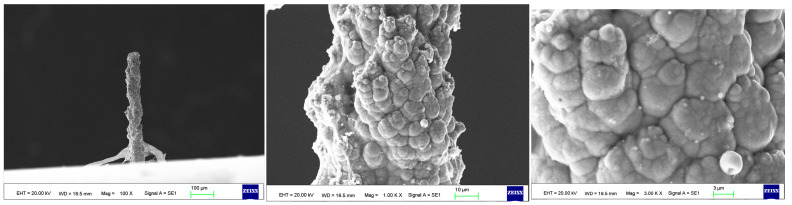
Surface morphology of micro-nickel column fabricated under anti-parallel magnetic field.

As the surface roughness distribution of the micro-nickel column is uneven and limited by the size of the fabricated micro-nickel column, a scanning electron microscope (SEM) was used to investigate the effect of external magnetic field on surface morphology of micro-nickel columns which fabricated with MLED. After cleaning and drying the sample with anhydrous ethanol, then observe it using a tungsten filament lamp SEM (EVO18, ZEISS, Jena, Germany). Figure 6 showcases the surface morphology (SEM) of micro-nickel column fabricated without magnetic field during MLED process. Figure 7 and Figure 8 showcase the microscopic surface morphology (SEM) of MLED micro-nickel columns fabricated under the influence of anti-parallel and parallel magnetic fields, respectively. In Figure 6, the micro-nickel column under SEM 100× exhibits an uneven diameter, with many cellular protrusions on the surface and a high degree of protrusion; at 1000× magnification under SEM, each large cellular protrusion contains several small cellular protrusions. Escalating the magnification to 2000×, each small cellular protrusion contains multiple small grain clusters, with uneven grain sizes and certain gaps between the grain clusters. In Figure 7, the micro-nickel column exhibits a uniform diameter; at 1000× magnification under SEM, the surface reveals spherical shaped block clusters, albeit with a low degree of protrusion. Escalating the magnification to 2000×, it becomes evident that small particles are congregated within each cytosolic cluster, displaying a uniform particle size and narrow inter-cluster gaps. Conversely, Figure 8 reveals a comparatively diminished morphological quality of the micro-nickel column, characterized by dendritic bifurcations on the surface, lacking in steepness and straightness. A closer examination of this micro-Ni column’s surface at higher magnification uncovers larger block clusters in a state of coalescence. Additionally, the microscopic surface manifests conical bifurcations aligned with the magnetic field direction, with nickel particles diminishing in size as one approaches the bifurcation tip.

From the above comparison, it can be seen that applying external magnetic field has an improvement effect on the micro surface morphology of MLED micro-nickel columns, and the effect of parallel magnetic field is better than that of anti-parallel magnetic field. The relative degradation of the surface quality of the micro-nickel columns fabricated under anti-parallel magnetic field is mainly due to the following reasons:

1. Exhibiting a high aspect ratio structure, the micro-nickel column remains upright and undistorted under its own weight, indicative of its relative stiffness in comparison to its weight. This phenomenon occurs as, at smaller length scales, body forces prove less effective in deforming structures than surface forces [27]. Nonetheless, the surface force holds less sway in comparison to the electric field force, resulting in the overall morphology of the micro-nickel column being predominantly influenced by the electric field force. However, during the deposition process, the tip portion of the column, constituting a smaller fraction of the overall structure, becomes more susceptible to the Lorentz force.
2. The Lorentz force propels the nickel ions upward, aligning with the direction of the magnetic field, where upon under the influence of the electric field, a reduction reaction ensues, culminating in the formation of nickel atoms. These atoms subsequently deposit in a layer-by-layer island fashion upon each particle cluster, exhibiting a progressive diminution in particle size. Theoretically, Faraday’s law posits the absence of Lorentz force generation in the context of a parallel magnetic field absent magnetic field and current interaction. Nevertheless, the micro-nickel columns being prone to magnetization, and their coupling with the electric field, induce anisotropic magnetic fields. Additionally, the growth of the micro-nickel columns distorts the electric field lines, engendering micro-scale MHD effects and consequently directing the magnetic metal atoms to grow along specific orientations during the electrodeposition process.
3. The implementation of pulsed electrodeposition alongside anodic microtips introduces ultra-high overpotentials to the deposition procedure. Concurrently, the evolution of hydrogen disrupts the nickel deposition, culminating in a surface with less uniformity.

### 4.3. Effect of Magnetic Field on Grain Size of MLED 3D Microstructure

**Figure 9:**
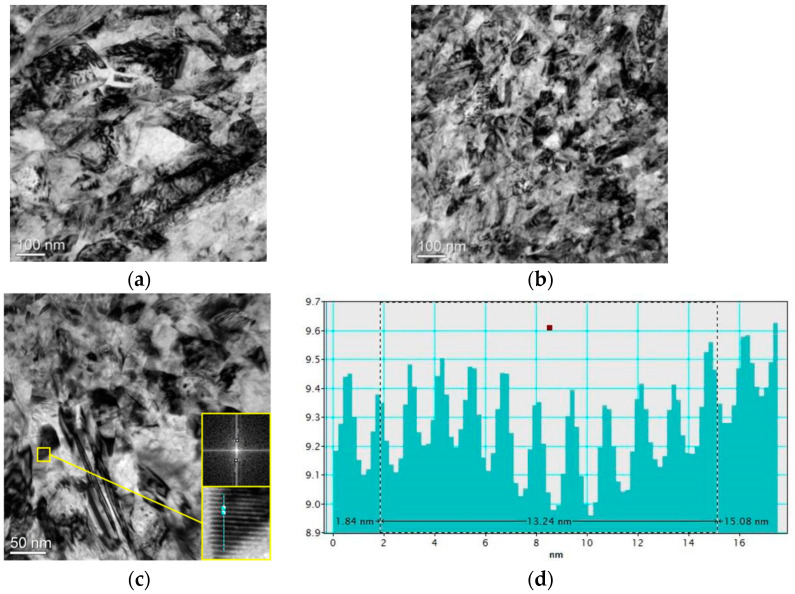
TEM images of nickel grain and crystal plane spacing bar graphs under action of external magnetic field on MLED micro-nickel columns: (**a**) action of parallel magnetic field (50,000×); (**b**) action of anti-parallel magnetic field (50,000×); (**c**) action of anti-parallel magnetic field (100,000×); (**d**) crystal plane spacing bar graphs under action of anti-parallel magnetic field.

A transmission electron microscope (TEM) was used to investigate the effect of external magnetic field on grain size of micro-nickel columns which fabricated with MLED. Using a focused ion beam (Scios 2 DualBeam, ThermoFisher Scientific, Waltham, MA, USA) to prepare TEM samples, and then observes them by the TEM (Tecnai F30, Philips FEI, Hillsboro, OR, USA). TEM images of nickel grain and crystal surface spacing bar graphs of MLED micro-nickel columns fabricated under different orientations of magnetic field are shown in Figure 9. Observations reveal that the grain size in Figure 9a exceeds that in Figure 9. TEM images of nickel grain fabricated under different orientations of magnetic field and crystal surface spacing bar graphs under action of parallel magnetic field of MLED micro-nickel columns, with smaller grain boundaries aggregating adjacent to larger ones. In contrast, Figure 9b displays a more uniform overall grain size, devoid of notable clustering. Figure 9c provides an expanded view of the cross sections of the micro-nickel columns fabricated under action of anti-parallel magnetic field. Here, the grain size appears smaller with blurrier grain boundaries in Figure 9c. Such blurring of grain boundaries signifies a higher energy demand for grain deformation and displacement, affirming that the micro-nickel columns fabricated under the influence of anti-parallel magnetic fields exhibit smaller grain sizes and superior internal performance [10]. Fourier transform analysis, conducted using Digital Micrograh software (3.9.1), facilitated the acquisition of diffraction patterns, which were then calibrated to ascertain the corresponding crystal plane spacings. Inverse Fourier transformation and the selection of a line segment perpendicular to the lattice stripes yielded bar chart of the respective distances (shown in Figure 9d), enabling crystallographic spacing measurements. The average crystal plane spacing, deduced from Figure 9d, stands at 1.32 nm, indicative of a preferential (110) orientation. This stems from the face-centered cubic (FCC) structure of nickel crystals, which inherently possess a more readily magnetizable [110] axis. To minimize free energy, paramagnetic *Ni* atoms exhibit a propensity to align with the magnetic field direction during the MLED process, thereby fostering the orientation of nickel crystals towards the (110) direction.

**Figure 10:**
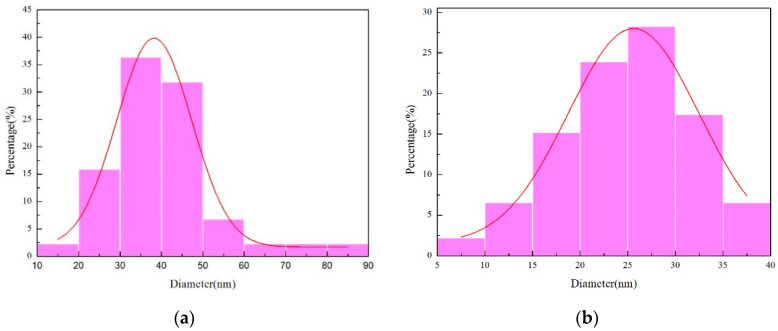
Chart of grain size distribution (**a**) parallel magnetic field; (**b**) anti-parallel magnetic field.

Figure 10 illustrates the outcomes of the grain size measurements analysis conducted using Nano Measurer software (V1.2). As depicted in Figure 10a, the grain size distribution of the micro-nickel columns fabricated under a parallel magnetic field exhibits a substantial variance, presenting an average grain size of 40.69 nm. The discrepancy between the smallest grain size (14.10 nm) and the largest grain size (85.30 nm) registers at 71.2 nm. In stark contrast, Figure 10b demonstrates a more uniform grain size distribution, yielding an average grain size of 24.82 nm and a discrepancy of 31.12 nm between the minimum grain size (8.01 nm) and the maximum grain size (39.13 nm). From these observations, it is inferred that the micro-nickel column fabricated under an anti-parallel magnetic field possesses a smaller grain size.

For this experiment, the value of pulse current is 1.8 mA, duty cycle is 0.3 and frequency is 10 kHz. Namely a low pulse current duty cycle, specifically a comparatively prolonged t_off_ time was established. The intention behind this configuration is to facilitate stress relaxation during t_off_, thereby interrupting the grain growth, and allowing the deposited ions to relax, which consequently leads to grain re-nucleation [28]. Concurrently, the incorporation of an external magnetic field introduces additional energy, enhancing the subcooling for nucleation and mitigating the stacking of atoms. This results in an escalated nucleation rate. In the deposition’s initial phase, a profusion of nickel nuclei materializes on the copper surface, with the nucleation pace surpassing the nuclear growth rate at this juncture. To sustain this condition, the external magnetic field continues to thwart atom stacking, diminishing the nuclear size. The reduction in crystal size facilitates a more balanced growth environment, bringing the crystal growth morphology closer to its equilibrium form. Establishing a balanced competition between crystal nucleation and growth is imperative for the successful fabrication of nanoscale grain microstructures [4].

## 5. Conclusions

The MLED process enables the fabrication of 3D microstructures featuring specific morphologies and nanoscale crystals, accomplished through meticulous control of plating parameters, precise regulation of additives, and the application of external force fields, all achieved without the need for templates. Compared with the existing literature of MLED, the experimental findings and test results presented in this paper elucidate that:

1. The implementation of a magnetic field markedly improves the fabrication rate of the MLED process, exhibiting enhanced efficiency under an anti-parallel magnetic field configuration. Concurrently, it influences the nucleation and grain growth within the deposited structure, yielding a microstructure characterized by nano-scale grains.
2. The magnetic field magnetizes the paramagnetic metal, inducing a micromagnetic field that influences the microscopic surface morphology of the three-dimensional microstructure. Observations also reveal that smaller grain sizes correlate with larger colonies exhibiting particle agglomeration and more distinct gaps between the colonies.
3. Although the magnetic field introduces substantial energy to the MLED deposition process, influencing the microstructure’s surface morphology, the anti-parallel magnetic field proves more advantageous for 3D microstructure preparation, considering the deposition rate and internal grain structure.
4. The MLED process involves complex interactions among magnetic fields, electric fields, flow fields, and other multiphysics fields, with mechanisms of action between them that remain obscure. Utilizing finite element analysis software such as COMSOL (V4.1a) and ANSYS (15.0) is considered to assist in elucidating these interactions.

## Data Availability

Data are contained within the article.
